# Supplementary figures and images for: Chlamydial protease-like activity factor targets SLC7A11 for degradation to induce ferroptosis and facilitate progeny releases
Source: PLoS Pathog. 2025 Apr 8;21(4):e1013060. doi: 10.1371/journal.ppat.1013060 (PMC12011302; doi:10.1371/journal.ppat.1013060)

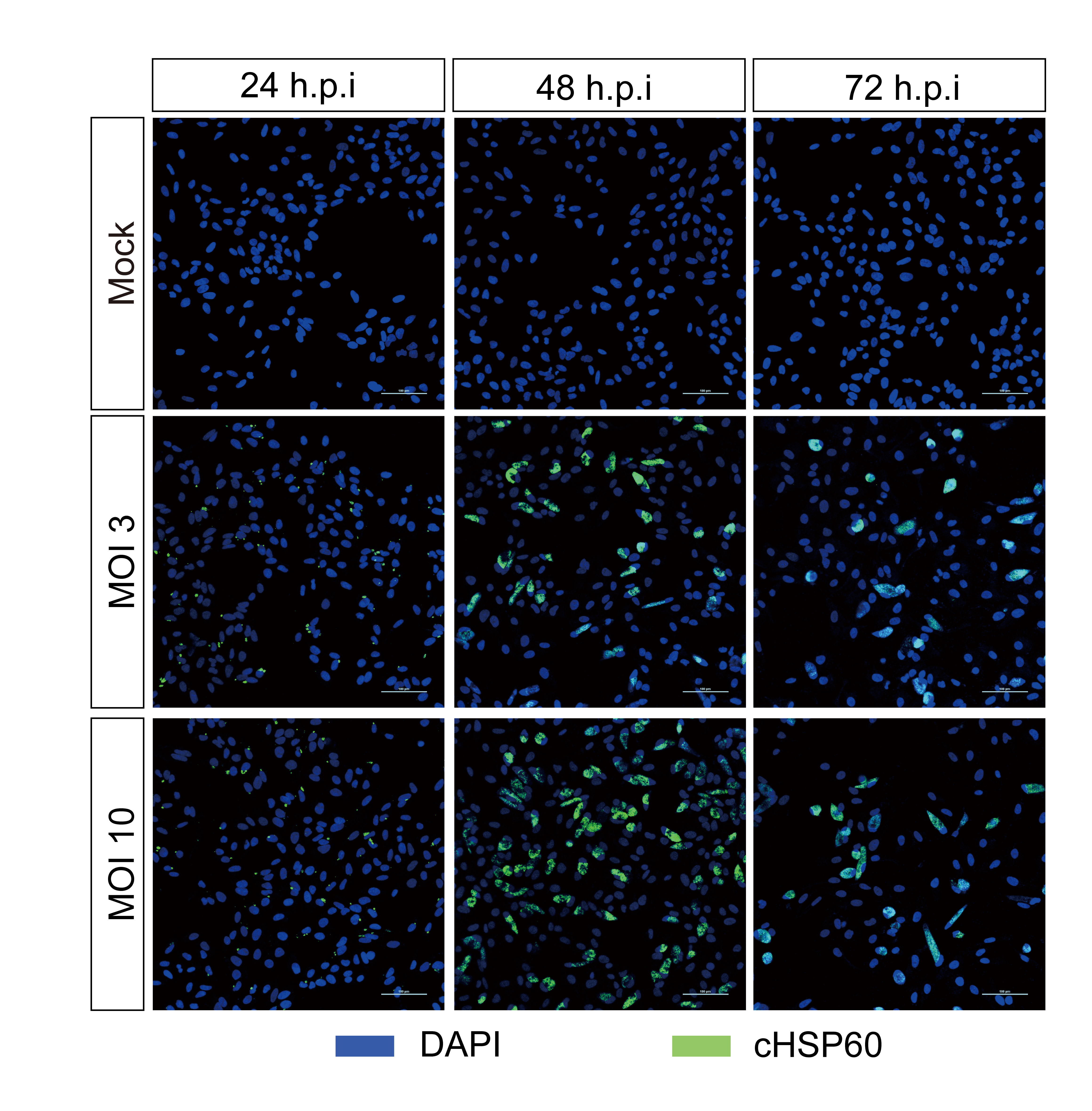

Supplement: S1 Fig — Immunofluorescence analysis of chlamydial HSP60 in Chlamydia trachomatis serotype D-infected cells was performed over a time course. (TIF) [file ppat.1013060.s001.tif]

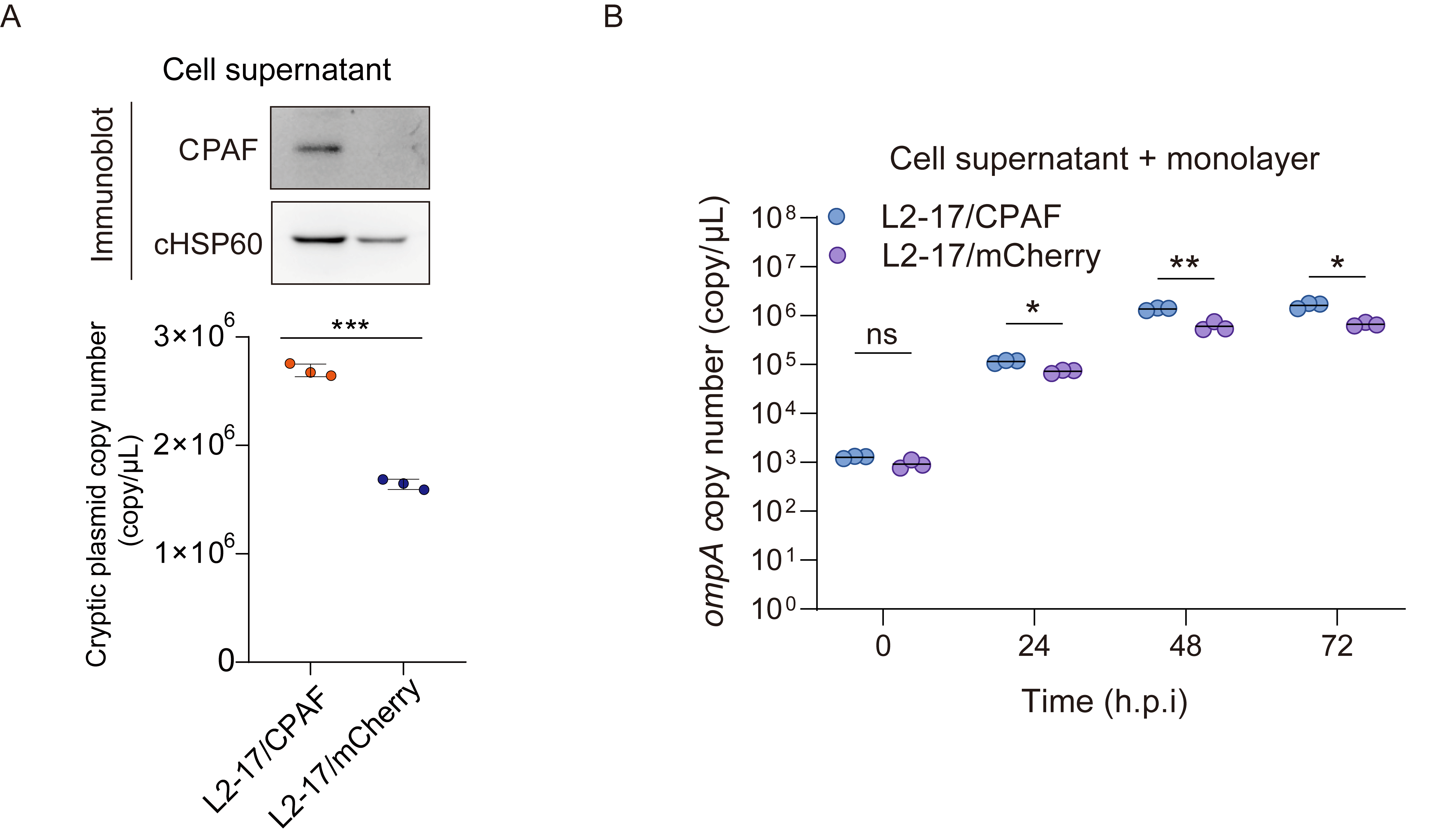

Supplement: S2 Fig — (A) The cryptic plasmid copy number and chlamydial HSP60 levels in the cell supernatant of CPAF-deficient strain (L2-17/mCherry) (MOI 1)-infected HeLa-229 cells were measured, compared to those in CPAF-supplemented strain (L2-17/CPAF) (MOI 1)-infected cells. Statistical analysis was performed using a Student’s t-test (n=3). (B) The ompA copy number in the total culture (supernatant and monolayer) of CPAF-deficient strain (L2-17/mCherry) (MOI 1)- or CPAF-supplemented strain (L2-17/CPAF) (MOI 1)-infected HeLa-229 cells was determined over a time course. Statistical analysis was performed using a two-way ANOVA (n=3). P values are indicated as follows: *, P < 0.05; **, P < 0.01; ***, P < 0.001. (TIF) [file ppat.1013060.s002.tif]

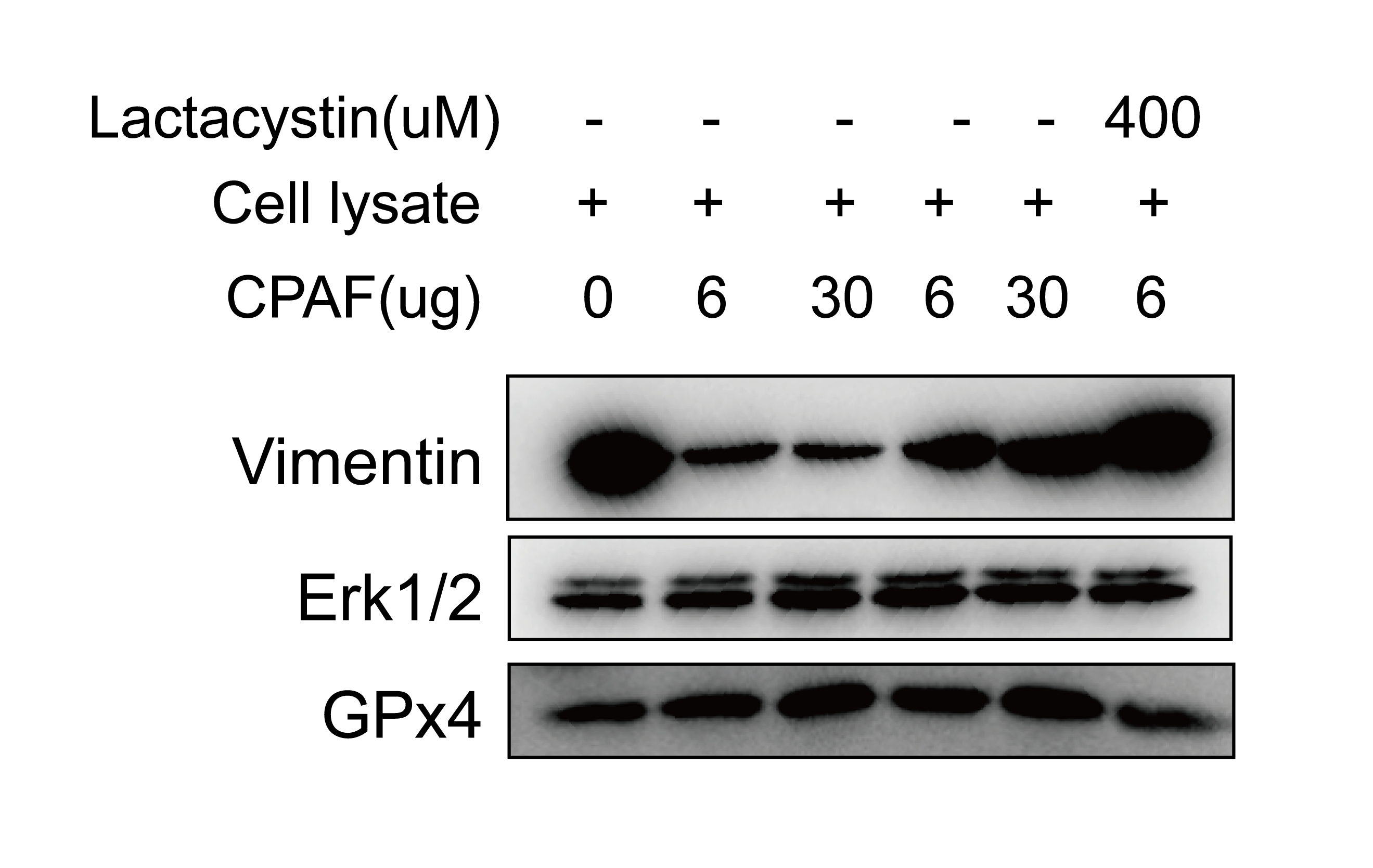

Supplement: S3 Fig — Cell lysates were incubated with recombinant wild-type CPAF and rCPAF(H105A) for 30 minutes. GPx4 was not degraded by rCPAF. Vimentin, a known CPAF post-lysis substrate, and ERK, a non-CPAF substrate, served as positive and negative controls, respectively. (TIF) [file ppat.1013060.s003.tif]
