# Supplementary material for: Chlamydial protease-like activity factor targets SLC7A11 for degradation to induce ferroptosis and facilitate progeny releases
Source: PLoS Pathog. 2025 Apr 8;21(4):e1013060. doi: 10.1371/journal.ppat.1013060 (PMC12011302; doi:10.1371/journal.ppat.1013060)
Supplement: Attachment — Submitted filename: Response_Letter.docx [file ppat.1013060.s006.docx]

**Response letter**

Dear Editors and Reviewers,

Thank you for your evaluation of our manuscript. We appreciate your constructive comments, which have greatly contributed to improving the manuscript. We have made every effort to address the reviewers’ concerns and have revised the manuscript accordingly. Below, we provide point-by-point responses to the editors' and reviewers' comments. Please note that the line numbers refer to the unmarked version of the revised manuscript.

**Editors’ comments:**

In light of the reviews (below this email), we would like to invite the resubmission of a significantly-revised version that takes into account the reviewers' comments. Particularly, the following two major issues must be addressed experimentally:

1. It is difficult to differentiate if the degradation of SLC is an artifactual processing of SLC in dead cells or during the sample processing by the released CPAF. The authors must be aware that several putative substrates of CPAF have subsequently been shown to be artifacts ([https://doi.org/10.1371/journal.ppat.1002842](https://jpn01.safelinks.protection.outlook.com/?url=https%3A%2F%2Fdoi.org%2F10.1371%2Fjournal.ppat.1002842&data=05%7C02%7C%7C33720fbccdc547f6f36e08dca6410aa5%7C84df9e7fe9f640afb435aaaaaaaaaaaa%7C1%7C0%7C638568045501530122%7CUnknown%7CTWFpbGZsb3d8eyJWIjoiMC4wLjAwMDAiLCJQIjoiV2luMzIiLCJBTiI6Ik1haWwiLCJXVCI6Mn0%3D%7C0%7C%7C%7C&sdata=1trmDb0FF08HVYYNNJ9PGzxW8oPs6UNV3sz9AAsl2cI%3D&reserved=0); Doi: [10.1016/j.micinf.2013.09.008](https://jpn01.safelinks.protection.outlook.com/?url=https%3A%2F%2Fdoi.org%2F10.1016%252Fj.micinf.2013.09.008&data=05%7C02%7C%7C33720fbccdc547f6f36e08dca6410aa5%7C84df9e7fe9f640afb435aaaaaaaaaaaa%7C1%7C0%7C638568045501549253%7CUnknown%7CTWFpbGZsb3d8eyJWIjoiMC4wLjAwMDAiLCJQIjoiV2luMzIiLCJBTiI6Ik1haWwiLCJXVCI6Mn0%3D%7C0%7C%7C%7C&sdata=7qyNNZFUn1SjgfJzImq73uWh9Z1iXvhrVhLKV3GH3DE%3D&reserved=0)). Thus, efforts must be made to ensure SLC processing occurs inside live cells or cells undergoing ferroptosis. Experimental conditions for excluding CPAF-mediated artificial processing can be found in this paper (doi: [10.1128/JB.02087-12](https://jpn01.safelinks.protection.outlook.com/?url=https%3A%2F%2Fdoi.org%2F10.1128%252FJB.02087-12&data=05%7C02%7C%7C33720fbccdc547f6f36e08dca6410aa5%7C84df9e7fe9f640afb435aaaaaaaaaaaa%7C1%7C0%7C638568045501563857%7CUnknown%7CTWFpbGZsb3d8eyJWIjoiMC4wLjAwMDAiLCJQIjoiV2luMzIiLCJBTiI6Ik1haWwiLCJXVCI6Mn0%3D%7C0%7C%7C%7C&sdata=Uu9LYqySINSK99OB56xjjqgPs3UT8tK9%2FVTy6ZioCFo%3D&reserved=0)), which should be applicable here.

***Response****: We sincerely appreciate these constructive comments. In response to this concern, we have conducted additional experiments to evaluate SLC7A11 following the recommended protocol (hot SDS buffer). The relevant results and corresponding modifications have been provided in detail below and incorporated into the revised manuscript.*

2. There are several concerns about the vitamin E animal data. First, medroxyprogesterone, a long-lasting progesterone, is typically used to synchronize the estrus cycle and sensitize mice to chlamydiae. However, the authors used regular progesterone, which has a half-life of less than an hour in mice. Is this a typo? The detected copy numbers are low for successful C. muridarum (CM) infection. Were the mice infected with CT instead of CM? Regardless, providing recoverable IFU data in vaginal swabs would be more convincing. A hallmark of successful CM infection is the formation of hydrosalpinx. Where are the hydrosalpinx data? The statement “CM-infected mice treated with vitamin E exhibited reduced dilation of the uterine horns (Fig. 4C)” is not supported by the pictures. It is important to demonstrate vitamin E's protective effect using macroscopic data (diameter of uterine horns, frequency of hydrosalpinx) and microscopic data (grades of inflammation and fibrosis). Examples for appropriately presenting chlamydia-induced pathology data can be found in references: [https://doi.org/10.1371/journal.pone.0095076](https://jpn01.safelinks.protection.outlook.com/?url=https%3A%2F%2Fdoi.org%2F10.1371%2Fjournal.pone.0095076&data=05%7C02%7C%7C33720fbccdc547f6f36e08dca6410aa5%7C84df9e7fe9f640afb435aaaaaaaaaaaa%7C1%7C0%7C638568045501577337%7CUnknown%7CTWFpbGZsb3d8eyJWIjoiMC4wLjAwMDAiLCJQIjoiV2luMzIiLCJBTiI6Ik1haWwiLCJXVCI6Mn0%3D%7C0%7C%7C%7C&sdata=vym4muieDptUDrPibqpKN0YIRbuU%2BHxnscCXxlwI1Ng%3D&reserved=0) and doi: [10.1128/IAI.01171-15](https://jpn01.safelinks.protection.outlook.com/?url=https%3A%2F%2Fdoi.org%2F10.1128%252FIAI.01171-15&data=05%7C02%7C%7C33720fbccdc547f6f36e08dca6410aa5%7C84df9e7fe9f640afb435aaaaaaaaaaaa%7C1%7C0%7C638568045501589630%7CUnknown%7CTWFpbGZsb3d8eyJWIjoiMC4wLjAwMDAiLCJQIjoiV2luMzIiLCJBTiI6Ik1haWwiLCJXVCI6Mn0%3D%7C0%7C%7C%7C&sdata=5y9Ffl2fkGzDx%2FlV4gTxAKmHoBRpgEn8tr5zUa%2FmTC4%3D&reserved=0).

***Response***: *Thank you very much for your valuable suggestions. Based on the references you recommended, we have repeated the animal experiments and corresponding microscopic analysis. The experimental results have been organized and visualized according to the references you provided, and the manuscript has been revised accordingly at the relevant sections.*

3. The three reviewers have pointed out many imprecise, incorrect, or contradictory/conflicting sentences throughout the manuscript. Please address them.

***Response***: *We sincerely appreciate Reviewer’s careful reading and valuable feedback. In response to this concern, we have thoroughly reviewed the entire manuscript and corrected imprecise, incorrect, or contradictory statements. We hope that these revisions have improved the overall quality and readability of the paper.*

**Responses to Reviewer's Questions**

**Part I - Summary**
Please use this section to discuss strengths/weaknesses of study, novelty/significance, general execution and scholarship.

Reviewer #1: Chen et al. investigated the mechanism underlying the exit of the obligate intracellular bacterium Chlamydia trachomatis from infected host cells at the end of its developmental cycle. Specifically, the authors tested the hypothesis that Chlamydia trachomatis activates ferroptosis through its secreted protease CPAF. While data from cell culture experiments appear to support this novel mechanism, there are notable weakness with critical data, including CPAF-mediated SLC7A11 in cell culture and the effect of vitamin E in animals. The study can be strengthened by addressing several issues.

***Response****: We sincerely appreciate Reviewer’s comments, which have greatly contributed to improving the quality of this study. We have made every effort to address these issues, and detailed responses are provided below for each specific comment.*

Reviewer #2: This manuscript by Chen et.al. entitled “Chlamydial protease-like activating factor targets SLC7A11 for degradation to induce ferroptosis and facilitate dissemination” describes a novel mechanism by which Chlamydia exits cells during the developmental cycle. The in vitro experiments are well designed and executed, for the most part, whereas the in vivo experimentation is somewhat rudimentary. The nomenclature and interpretations are somewhat overstated. Addressing some issues listed below might improve the manuscript.

***Response***: *We sincerely thank Reviewer’s comments. In response, we have repeated the in vivo experiments to strengthen the article. Also, we have revised the manuscript to ensure that the nomenclature and interpretations are more accurately presented. Detailed responses to the specific comments are provided below.*

Reviewer #3: In this interesting paper the authors propose that the chlamydia serine protease CPAF is degrading the host glutamate-cystine antiporter SLC7A11 (SLC) to drive ferroptopsis and promote the release of chlamydial infectious particles (EBs). The paper is clearly written, the experiments that were performed seem solid, and I really enjoyed the clear model presented in 4F. That stated, this paper has several critical limitations that make it difficult to differentiate if their model is correct or if the degradation of SLC that is being observed is an artifactual processing of SLC in dead cells by CPAF released from burst inclusions. Notably, several putative substrates of CPAF have subsequently been shown to be artifacts via the explanation I just proposed, and this manuscript seems to have incompletely considered this data and did not control several experiments sufficiently to differentiate. In particular, I had issues with the absence of one step growth curves in several places. This would have allowed me to assess if the treatments were having the specific effects the authors reported or more likely reflected differences due to growth rates. Second, and related to this, the progeny assays that were performed only looks at the supernatants; again, if chlamydia grew more slowly in the compared conditions just looking at the supernatant could yield misleading results. Finally, and most importantly, no data was shown that proved to me that SLC was being processed inside intact cells. Out of all of my concerns, addressing the last point is absolutely critical to justify the conclusions that were presented.

***Response****：We thank Reviewer for their constructive comments. In response, we have conducted additional experiments, including Chlamydia growth curves following treatment with several pharmaceutical inhibitors and Chlamydia growth curves of CPAF-deficient and CPAF-complemented strains. We also evaluated SLC7A11 by blocking the post-lysis effect of CPAF using hot SDS buffer. Detailed responses to specific comments are provided below.*

Specific comments
Line 57. Clarify that this organism is the cause of chlamydia (which is the most common bacterial STI) and lymphogranuloma venereum.

***Response****: We have clarified in lines 57-58.*

Sentence starting line 59: It is unclear how PID and these other outcomes are key to chlamydial dissemination and propagation. These disease manifestations could be detrimental to chlamydia, unaware of data that support either possibility.

***Response****：Thanks. We agree with Reviewer’s comments. We have removed this statement.*

Sentence starting line 65: It is not clear if lysis is the only method progeny are released in vivo. Extrusion is ignored, and, notably, this could be a limitation of interpretation of progeny assays employed later in this study.

***Response****: Thank you. We have introduced both extrusion and lysis mechanisms in the introduction section (lines 63-69) and stated the following in lines 496-499 of the revised manuscript: “Although the Chlamydia-infected cell supernatant we detected contained progeny released via both lysis and extrusion approaches, pharmacological inhibition of ferroptosis successfully reduced progeny release, suggesting the important role of ferroptosis in this process.”*

Line 85: The idea that CPAF is secreted from intact inclusions is wildly controversial. Many of the cited papers have essentially been retracted. It is clear that CPAF can cleave a wide variety of host cell substrates after lysis of the inclusion. This whole paragraph needs to be heavily caveated with the insights from the Tan group’s work (see PMID: 22876181 for example).

***Response****: We sincerely appreciate Reviewer’s comments. We have reviewed the work of Tan's group and removed the retracted papers from the citations, revising the manuscript accordingly.*

Line 262: To get an idea of the kinetics of chlamydial development using the criteria you describe it is absolutely essential that one-step growth curves be performed. A major concern I have about the entire paper is that authors are looking at what is going on 72 h post infection at high MOIs in cycloheximide treated cells. In my experience, every cell in the flasks is going to be heavily damaged by that point.

***Response:*** *We thank Reviewer for the comments. To address this question, we have provided an IFA image showing Chlamydia growth in Fig. S1. At 72 h.p.i., the inclusions in the monolayer were significantly fewer than at 48 h.p.i.*

*We apologize for the oversight in not clearly specifying the use of cycloheximide in the submitted manuscript. Cycloheximide was only applied during the ferrostatin-1, liproxstatin-1, and trolox treatment experiment (Fig.2B-J in the revised manuscript). This clarification has now been added on lines 114-116.*

Figure 1A: I can tell what is going on from the image, I assume the small specks are PI positive? Does not add much.

***Response:*** *We have provided high-quality images in Fig. 1A, where PI-positive cells are clearly visible. The infectivity and virulence of Chlamydia strains can vary between laboratories. We have confirmed that Chlamydia, including different serotypes and mouse-adapted strains, induces cell lysis during the late stages of infection. This conclusion is consistent with findings from several published studies (Kevin Hybiske and Richard S. Stephens, Proceedings of the National Academy of Sciences,2007).*

Line 270-284: My interpretation is here that you have a very subtle increase in ROS that occurs very late in heavily infected cultures, and that inhibitors of ferroptosis have a complementary but very minor effect around the same time point.

***Response****: We thank Reviewer for the comments. The observations of increased lipid ROS were quantified across several Chlamydia serotypes and align with the findings by Azenabor AA and Mahony JB (Int J Infect Dis, 2000). In our study, we used several ferroptosis inhibitors to demonstrate the role of ferroptosis in this process, focusing on their capacity to reduce lipid ROS levels and Chlamydia-triggered cell death (Fig.2).*

Paragraph starting line 285: The experiments are not able to differentiate if “inhibition of ferroptosis could effectively attenuate the release of CT progeny” from if the inhibitors caused CT to develop more slowly. This could have been addressed by looking at total genomes and or progeny in the remaining cells as well as supernatant. This issue presents in several subsequent figures and is a significant limitation.

***Response****: Thank you for your comments. To address this concern, we have quantified the ompA copy number across three ferroptosis inhibitors (ferrostatin-1, liproxstatin-1, Trolox), and the results are presented in* ***Fig. 2E*** *and* ***2J****. However, only ferrostatin-1 demonstrated an effect on Chlamydia growth after 72 hours of treatment, while the other inhibitors did not affect Chlamydia growth. Consequently, we proceeded with liproxstatin-1 for further experiments(****Fig.2F-G****). All of the results support our conclusion that inhibition of ferroptosis can attenuate progeny release.*

*We have revised the manuscript as follows: “To exclude the possibility that the reduction in progeny levels in the supernatant was due to a direct inhibitory effect of the drugs on Chlamydia growth, we monitored the growth dynamics of Chlamydia under drug treatment compared to the control group. The results showed that neither ferrostatin-1 nor liproxstatin-1 significantly inhibited Chlamydia growth, except for ferrostatin-1, which demonstrated an inhibitory effect after 72 hours of treatment. (****Fig. 2E****).”* *Additionally, “the potential for trolox-induced inhibition of Chlamydia growth was ruled out, as confirmed in* ***Fig. 2J****.” These revisions are now reflected in lines 302-307 and 314-315, respectively.*

Paragraph starting line 294: The western blotting results could just as easily be explained by the degradation of SLC in lysed cells, by CPAF released from lysed inclusions. Again, this experiment is being done very late during infection, there are lots of burst inclusions and CPAF floating around. This could have been tested by clarifying if there were lower levels of intact SLF in cells containing intact inclusions prior to inclusion lysis. Alternately, the blot could have included antibodies for other plasma membrane proteins that CPAF can cleave in vitro but not in vivo.

***Response****: Thanks. According to previous reports, cell lysed by hot SDS buffer could denature CPAF, which helps exclude the potential CPAF that might remain during cell lysis. Therefore, we conducted an experiment to evaluate SLC7A11 over a time-course by using hot SDS buffer lysis protocol.*

*As shown in* ***Fig. 5F*** *of the revised manuscript, the degradation of SLC7A11 levels was consistently accompanied by an increase in CPAF levels from 24 to 72 h.p.i. Meanwhile, vimentin-previously identified as a CPAF substrate that undergoes degradation due to post-lysis effects-showed no change and served as experimental control. The SLC7A11 identified in this study also aligns with the dataset of CPAF substrates determined through a proteomic approach in a previous study (Patton MJ et al., mBio, 2016). Additionally, functional validation showed that depletion of SLC7A11 led to a significant reduction in intracellular GSH levels, which are imported via the xCT transporter formed by SLC7A11 and SLC3A2 in both CT-infected and CPAF-expressed cells. These results support the idea that SLC7A11 degradation is part of the infection process, not caused by post-lysis protease effects mediated by CPAF.*

*The relevant revision has been highlighted in lines 397-402 and 458-466 of the manuscript.*

Sentence starting line 317: Did lactacystin slow down growth or did it have the stated effect? With all of these experiments, where chlamydia is in development is critical for the comparison; ie if you slow development down there will be differing amounts of free CPAF released from lysed cells. BTW, CT expresses additional S41 family proteases, how do you know if it was Tsp or CPAF?

***Responses****: We sincerely thank Reviewer for their valuable comments. As outlined in the originally submitted manuscript, lactacystin treatment was administered at 66 hours post-infection for a brief duration of 6 hours. In response to concerns regarding the potential impact of lactacystin on Chlamydia growth, we quantified the total ompA gene copy number (including both the supernatant and cultured cells) in the DMSO and lactacystin treatment groups, both before and after lactacystin treatment.*

*In the revised of manuscript, we stated that “We also excluded the potential inhibition of Chlamydia growth by lactacystin (****Fig. 5C****).” in lines 371-372.*

*To address Reviewer’s question regarding Tsp, we have discussed as follows: “Our study did not prioritize other Chlamydia proteases, such as Tsp, because previous research has shown that overexpression of Tsp inhibits Chlamydia development, and other reports have demonstrated that Tsp is detected only in the inclusion, rather than the cell cytosol [48, 49]. We speculate that Tsp targets chlamydial rather than host proteins, and that excessive Tsp may hinder progeny release. Moreover, no studies have suggested that Tsp regulates cell death or participates in Chlamydia release during the late stages of infection. Of course, this does not exclude the possibility that Tsp might be involved in this process.” in lines 486-493 of revised manuscript.*

Paragraph starting line 323: Consider all of my comments above and what would happen if the CPAF null and complement strains develop with slightly different kinetics? One step growth curves and or progeny assays that count organisms in the monolayer and free in the sup simultaneously could be used to differentiate this critical question.

***Responses****: We sincerely appreciate Reviewer’s insightful and constructive comments. In response to the suggestion, we have quantified the growth curves of both the CPAF-deficient (L2-17/mCherry) and complemented (L2-17/CPAF) strains. The CPAF-deficient strain exhibited attenuated growth kinetics, as illustrated in* ***Fig.S2****. This finding is consistent with previous studies, which have shown that C. trachomatis CPAF-null mutants exhibit a reduction in the generation of infectious progeny (Frontiers in Cellular and Infection Microbiology, 2018). Therefore, the reduced amount of chlamydial progeny in the culture supernatant, compared to the complemented strain, may result from both the impaired ability to generate infectious progeny and the decreased efficiency of the lytic process in releasing infectious progeny.*

*In lines 479-484, we have state as follows:* “…*due to the complexity of the context, where CPAF-deficient strains exhibit weaker growth kinetics compared to CPAF-complemented strains across multiple studies [28, 47]. It is difficult to determine whether the inability to induce ferroptosis limits progeny release, or if the reduction in progeny production is due to CPAF deficiency, or whether a weakened resistance to immune clearance is at play, or a combination of these factors.”*

Line 431: As the authors’ own data shows, there are lot of dead and lysed cells in the culture by 72 hpi. For the urea experiment to show anything a time-course would be needed. If secreted CPAF is degrading SLC inside intact chlamydia infected cells, urea would make no difference. I suspect is such a time course were performed the authors would see that the onset of SLC degradation was concomitant with the initiation of progeny production. Separately, IFA would show no evidence of SLC degradation inside cells with intact inclusions.

***Response****: We sincerely thank Reviewer for their constructive comments. We fully agree with Reviewer’s suggestion to conduct a time-course experiment and have subsequently implemented it. We evaluated SLC7A11 levels at multiple time points, from 24 to 72 hours post-infection, using the recommended protocol. As shown in* ***Fig.5F****, the degradation of SLC7A11 levels was consistently accompanied by an increase in CPAF levels over this period, while vimentin, previously identified as a CPAF substrate that undergoes degradation due to post-lysis effects, showed no change and served as an experimental control. The relevant revision has been highlighted in lines 397-402 of the manuscript.*

**Part II – Major Issues: Key Experiments Required for Acceptance**
Please use this section to detail the key new experiments or modifications of existing experiments that should be absolutely required to validate study conclusions.

Generally, there should be no more than 3 such required experiments or major modifications for a "Major Revision" recommendation. If more than 3 experiments are necessary to validate the study conclusions, then you are encouraged to recommend "Reject".

Reviewer #1: 1. The rationale for analyzing cell death at 48 and 72 hpi is unclear. It is well-established that with cycloheximide, most cells infected with C. muridarum, and to a lesser degree with C. trachomatis serovar D and L2, would have lysed by 48 hpi, while lysis in cycloheximide-free cultures is somewhat delayed. It has been stated that cycloheximide was used for the ferrostatin and liproxstatin-1 experiments. Was cycloheximide also used in other experiments?

***Response****: We thank Reviewer’s comments. As shown in* ***Fig. 1A-C****, we observed the significant death (from PI staining and LDH release assay) at 48 and 72 h.p.i in our experiment context. This observation was in line with previous reports that the maximal levels of cell death occur at 72 h (Kevin Hybiske and Richard S. Stephens, Proceedings of the National Academy of Sciences,2007).*

*We apologize for the oversight in not clearly specifying the use of cycloheximide in the submitted manuscript. We have clarified the use of cycloheximide in the methods section, specifically in lines 114-116: “Cycloheximide (1 μg/mL; MedChemExpress, Shanghai, China; HY-12320) was added to the culture to accelerate cell death induced by Chlamydia during the ferrostatin-1, liproxstatin-1, and trolox treatment experiment (Fig.2B-J).”*

2. Figures 1A and other images suggest extensive cell lysis at 48 and 72 hpi. However, upon closer inspection, most cells can still be seen. The quality of data can be improved by expanding the image size (e.g., use 2-full columns width for Fig. 1A), reacquiring images as phase-contrast if they are not, using shorter exposure times under white light for better cell visibility, and presenting a separate set of immunostained images showing inclusions with MOIs of 3 and 10 as supplemental data to document chlamydial growth clearly. Images in Figures 1D and 3A should be improved or replaced for the same reason.

*Response: We re-conducted the relative experiments according to Reviewer’s suggestions. High-quality images were provided in* ***Fig.1A****. A separate set of IFA images showing inclusions with MOIs of 3 and 10 was provided in the supplemental data (****Fig.S1****) to document chlamydial growth clearly. In our revised manuscript, we have reorganized the structure of the article and the figures. Considering space constraints, we have retained the LDH quantification as a measure of cell death in other experiments.*

3. Western blotting for SLC7A11 cleavage was done with RIPA buffer, which allows post-lysis cleavage. To confirm physiological cleavage in infected cells, use SDS-PAGE sample buffer (which is also often called SDS-PAGE gel loading buffer) with DTT and immediately heat samples to 100℃ to inactivate CPAF (e.g., Patton et al., 2018).

***Response****: We sincerely thank Reviewer for the suggestions. We have conducted the experiment using the recommended protocol. As shown in* ***Fig 5F****, SLC7A11 was significantly degraded during the later stages of infection, while vimentin, previously identified as a CPAF substrate that undergoes degradation due to post-lysis effects, exhibited no change and served as experimental control.*

*In lines 397-402, we have revised the manuscript as follows: “To confirm that CPAF-mediated degradation of SLC7A11 was not affected by post-lysis effects, we assessed SLC7A11 levels at multiple time points (24-72 h.p.i) using a protocol that blocks CPAF post-lysis effects [30]. As shown in* ***Fig. 5F****, SLC7A11 degradation was consistently accompanied by an increase in CPAF levels. In contrast, vimentin identified as a substrate undergoing CPAF post-lysis degradation remained unchanged, serving as an experimental control [26].”*

*In lines 457-460, we state that as follows: “Our results confirmed that SLC7A11 was processed by CPAF within the cell, eliminating the potential confounding effects of post-lysis processes. SLC7A11 identified in this study also matches the dataset of CPAF substrates (not functionally validated) determined through a proteomic approach in previous studies [27].”*

4. There are several issues with the data from the animal experiments. In the chlamydial mouse genital tract infection model, medroxyprogesterone, a long-lasting progesterone, is typically used to synchronize the estrus cycle and sensitize mice to chlamydiae. However, authors used regular progesterone, which has a half-life of less than an hour in mice. This is unlikely to have a lasting effect. The detected copy numbers seem low, raising the question of successful infection establishment. Providing recoverable IFU data in vaginal swabs would be more convincing. A hallmark of successful C. muridarum infection is the formation of hydrosalpinx. When repeating the study, present hydrosalpinx data. The statement “CM-infected mice treated with vitamin E exhibited reduced dilation of the uterine horns (Fig. 4C)” is not supported by the pictures. To convincingly demonstrate a protective effect of vitamin E, present both macroscopic data (diameter of uterine horns, frequency of hydrosalpinx) and microscopic data (grades of inflammation and fibrosis). Considering the variation in uterine horn thickness shown in Fig. 4C, there may be a need to increase the number of animals per group.

***Response****: We appreciate Reviewer’s comments and apologize for the limitations in the initial animal experiments. In response, we have followed the methods from previous studies to re-conduct the animal experiments. The updated results, including recoverable IFU data from vaginal swabs, hydrosalpinx data, and microscopic observations, are presented in* ***Fig. 3****. Additionally, we have increased the number of animals per group to 8, as requested. The animal experiments indicated that vitamin E reduces the Chlamydia burden in low genital tract of mice and trends toward attenuation of pathology, as mentioned in lines 319-341 of revised manuscript.*

5. P-values are from experiments with multiple groups. Have any statistical procedures been performed to control the false discovery rate?

***Response****: Thank you. We have clarified this in the methods section and figure legends. In lines 269-271, we state: “Statistical analyses are provided in the respective figure legends. For multiple comparisons (more than two groups), a Bonferroni correction was applied.”*

Reviewer #2:

1. Title: Chlamydia protease-like activity factor is generally the term described in literature. Please clarify the name.

***Response****: Thank you for pointing out this error. We have corrected it throughout the entire manuscript.*

2. Fig 1G: Whereas DMSO is used as positive control for necrosis and chlamydial release from cells, it would be appropriate to include mock as negative control for statistical comparisons.

***Response****: Thank you for Reviewer's comments. We respectfully believe that a mock group is not appropriate.* *The goal of our experiment in Fig. 1G of the originally submitted manuscript was to assess the impact of ferroptosis inhibition on Chlamydia release in both ferrostatin-1 and liproxstatin-1 treatment groups. We included a DMSO group as the vehicle control for comparison, which aligns with our experimental objectives, while the use of the mock group did not permit detection of Chlamydia.*

3. Fig 3: Dissemination is generally used to describe spread of organisms from one tissue/organ to others. A cell culture demonstration of bacterial release does not demonstrate dissemination. Descriptions should reflect data shown.

***Response*** *: Thank you for Reviewer's comments. We have revised the manuscript accordingly and now use the phrase 'the release of progeny' rather than 'dissemination' to ensure greater precision and appropriateness.*

4. Rationale for the study: The key rationale for this study is stated as the lack of cost-effectiveness of the currently licensed vaccines (Lines 79-82 and 88-91). The JCVI reference (#9) for this does not contain information supporting this statement. It is unclear how reference #12 supports this point of view. Additionally, there is no data described in this manuscript that demonstrated potential cost-effectiveness of the new vaccine being studied. Therefore, the manuscript does not present data to address the purported gap in knowledge that the current study is intended to address.

***Response****: We greatly appreciate Reviewer's feedback, but we are somewhat confused by these comments. In the original manuscript we submitted, we did not mention the lack of cost-effectiveness of the currently licensed vaccines. Additionally, the reference (#9) is from JCI, not JCVI. In lines 79-82 and 88-91 of the original submitted manuscript, we state: “Moreover, supplementing Chlamydia-infected lambs with vitamin E, a potent inhibitor of ferroptosis, results in improved treatment outcomes” and “This proteolytic activity allows CPAF to undermine host defense mechanisms, facilitate chlamydial survival and replication, and evade host immune surveillance. While CPAF has been implicated in the cell lysis process, the specific mechanism by which it operates remains poorly understood.” We respectfully suggest that this comment may not be directly related to the current manuscript.*

5. Fig 1E: Uterine horn chlamydial burden was only assessed on day 14. It would be important to show kinetics of bacterial burden in uterine horn. Perhaps the authors can comment whether they consider spread of organisms from lower genital tract to uterine horn as ascension or dissemination (involving spread via blood/lymph)?.

***Response****: Based on our findings that the inhibition of ferroptosis reduces progeny release, we hypothesized that ferroptosis inhibition would limit progeny release in situ, thereby impairing the establishment of new infections in the surrounding area and ultimately leading to a suppressed infection in vivo. To address this hypothesis, we have reconducted the animal experiment. Under this setup, Chlamydia detection in the uterine horn at 56 dpi is unlikely. Therefore, we focused more on the Chlamydia burden in the lower genital tract and the final pathology of the upper genital tract. The animal results indicated that inhibiting ferroptosis with vitamin E reduces the Chlamydia burden in the lower genital tract of mice and trends toward attenuation of pathology.*

6. Fig 4: whereas the title implicates CPAF in the described effects, no in vivo experimentation was conducted to demonstrate the same. The authors should consider ways to demonstrate how CPAF contributes to ferroptosis and chlamydial spread in vivo.

***Response:*** *We thank Reviewer’s comment. Previous studies have demonstrated that CPAF-deficient strains are cleared more quickly than wild-type strains in mice (Yang Z, et al. Infect Immun, 2016; Rajeeve K,et al. Nat Microbiol, 2018). Based on our in vitro experiments, which show that CPAF-deficient strains induce less cell death, and previous reports indicating a lower Chlamydia burden in the lower genital tract compared to the wild-type strain, it is intuitive to hypothesize that the ferroptosis phenomenon would be weaker in CPAF-deficient infections.*

*The reduced Chlamydia burden in the lower genital tract and the potentially weaker transmission of CPAF-deficient strains make it difficult to determine whether the inability to induce ferroptosis limits progeny release, or if the reduction in progeny production is due to CPAF deficiency, or whether a weakened resistance to immune clearance is at play, or a combination of these factors. We apologize for being unable to address these questions directly in this study. We acknowledge these limitations and have discussed them in lines 476-485.*

In summary, this manuscript describes a novel putative mechanism that chlamydia may employ to spread between tissues/organs. Whereas much of the in vitro experiments lend support to this, in vivo experiments do not fully connect the contribution of CPAF to observed effects.

***Response****: We thank Reviewer’s summary. As mentioned in our previous response, we have acknowledged the limitations of our study, particularly the inability to fully elucidate the contribution of CPAF deficiency in vivo. However, we cannot overlook that our in vitro experiments provide strong evidence supporting the potential role of CPAF in ferroptosis and Chlamydia progeny release. Future research will need to further investigate the specific role of CPAF in in vivo infection.*

Reviewer #3:

1. Several experiments would benefit from one step growth curves to show that different growth rates do not explain the given phenotypes.

***Response****: Thank you for your comments. We conducted Chlamydia growth curve experiments using several pharmacological inhibitors (ferrostatin-1, liproxstatin-1, Trolox, and lactacystin), and the results are presented in* ***Fig. 2E****,* ***2J****, and* ***5C****. None of the inhibitors affected Chlamydia growth, except for ferrostatin-1 treatment for 72 hours (****Fig. 2E****). Based on these findings, we chose liproxstatin-1 for further experiments, including testing with other Chlamydia serotypes (****Fig. 2F-G****). These results suggest that inhibiting ferroptosis can reduce the release of progeny without affecting Chlamydia growth.*

*In lines 305-307, we stated: “The results showed that neither ferrostatin-1 nor liproxstatin-1 significantly inhibited Chlamydia growth, except for ferrostatin-1, which demonstrated an inhibitory effect after 72 hours of treatment (****Fig. 2E****).”*

*We revised the manuscript as follows: “Additionally, the potential for trolox-induced inhibition of Chlamydia growth was ruled out, as confirmed in* ***Fig. 2J****.” and “We also excluded the potential inhibition of Chlamydia growth by lactacystin (****Fig. 5C****).” in un lines 314-315 and 371-372, respectively.*

*In lines 439-441 of the revised manuscript, we mention: “After controlling for potential Chlamydia growth inhibition effects, several ferroptosis inhibitors were found to reduce Chlamydia progeny release during the late stages of infection.”*

2. IFA or other evidence that SLC is being degraded inside infected cells that contain intact inclusions.

***Response****：As shown in* ***Fig. 5F*** *of the revised manuscript, the degradation of SLC7A11 levels was consistently accompanied by an increase in CPAF levels from 24 to 72 h.p.i. Additionally, from a functional validation perspective, the observation that depletion of SLC7A11 resulted in a significant reduction in intracellular GSH levels, which is imported into the cell via the xCT transporter formed by SLC7A11 and SLC3A2 in both CT-infected and CPAF-expressed cells, further supports the idea that the degradation of SLC7A11 was not caused by post-lysis protease effects mediated by CPAF. Instead, it suggests that the degradation of SLC7A11 is an integral part of the infection process itself.*

3. 8M urea experiments should be presented as a time course, with one-step growth curves in parallel. The key question is, does degradation of SLC one start before or after host cells lyse.

***Response****：Thank you. We conducted a time-course experiment using cells lysed with hot SDS buffer (a protocol that blocks the CPAF post-lysis effect). The immunoblot of CPAF in Fig. 5F and IFA in Fig. S1 indicate the Chlamydia growth status.*

*To address the key question, we have revised the manuscript in lines 397-402 as follows: “To confirm that CPAF-mediated degradation of SLC7A11 was not affected by post-lysis effects, we assessed SLC7A11 levels at multiple time points (24-72 h.p.i) using a protocol that blocks CPAF post-lysis effects [30]. As shown in* ***Fig. 5F****, SLC7A11 degradation was consistently accompanied by an increase in CPAF levels. In contrast, vimentin identified as a substrate undergoing CPAF post-lysis degradation remained unchanged, serving as an experimental control [26].”*

**Part III – Minor Issues: Editorial and Data Presentation Modifications**
Please use this section for editorial suggestions as well as relatively minor modifications of existing data that would enhance clarity.

Reviewer #1:

1. Figure legends should be more informative. For example, include the timing of ferrostatin-1 and liproxstatin-1 treatments.

***Response****: Thank you. We have revised all the figure legends.*

2. Increase the size of labels in most (and perhaps all) figures, particularly Fig. 3, as there is ample space to expand.

***Response****: We have reorganized the figures accordingly.*

3. Line 168: Specify the “green dye.

***Response****: The “Green Dye” is one of the components in the reagent kit, labeled as “Green Dye”. As specified in line 169, it is referred to as “Green Dye (Component A).”*

4. Line 229: Delete “buffer” before “After.”

***Response****: Thank you for pointing out the error. We have corrected it in line 242.*

5. Line 277: Change “identified” to “detected.”

***Response****: We have corrected it in line 286.*

Reviewer #2: (No Response)

***Response****: We thank Reviewer’s comments.*

Reviewer #3: See overall review.

***Response****: Thanks.*

*In closing, we wish to thank the PLOS Pathogens for considering our work and express our appreciation to the Reviewers for their effort and constructive comments.*
